# Supplementary material for: Subclass Analysis of Malignant, Inflammatory and Degenerative Pathologies Based on Multiple Timepoint FAPI-PET Acquisitions Using FAPI-02, FAPI-46 and FAPI-74
Source: Cancers (Basel). 2022 Oct 28;14(21):5301. doi: 10.3390/cancers14215301 (PMC9656977; doi:10.3390/cancers14215301)
Supplement: Supplementary file 1 [file cancers-14-05301-s001.zip › cancers-1946729-supplementary.pdf]

Subclass Analysis of Malignant, Inflammatory and Degenerative Pathologies Based on Multiple Timepoint FAPI-PET Acquisitions Using FAPI-02, FAPI-46 and FAPI-74

SUPPLEMENTAL MATERIAL

Supplemental Table S1: Uptake kinetics of other specific lesions not shown separately in the figures

^{68}Ga-FAPI-uptake (SUVmax and SUVmean) of specific types of lesions, that were not considered separately for the other analyses so far or did not match the other types of classifications, over time with acquisition timepoints 10 min (1), 22 min (2), 34 min (3), 46 min (4) and 58 min (5) p.i..

Tracer variant	Lesion	Standardized uptake value [g/ml]									
		1		2		3		4		5	
		max	Mean	max	mean	max	mean	max	mean	max	mean
FAPI-02	Angiomyolipoma of the left kidney	3,1759	2,2363	2,7382	2,0760	2,6426	2,0799	2,4667	1,9142	2,2961	1,8686
	Asbestosis of the right pleura	4,5687	2,9415	3,8508	2,6319	3,3975	2,2868	3,5824	2,4112	3,2380	2,3747
FAPI-46	Unspecific solid tumo	6,2072	4,1696	4,6019	3,4030	4,0166	2,9605	3,3426	2,5113	3,3649	2,4522

	ur of the right kidney										
	Oesophagitis; patient 1	2,9983	1,7290	2,4097	1,6562	2,5119	1,6936	2,7488	1,7022	2,4886	1,6702
	Oesophagitis; patient 2	7,8309	5,0977	8,4310	5,6800	10,224	5,6659	10,8621	6,3345	10,4263	7,2717
	Oesophagitis; average	5,4146	3,4130	5,4204	3,6681	6,3680	3,6798	6,8055	4,0184	6,4575	4,4710
	Spinous process (L3); patient 1	2,7944	1,9118	2,2133	1,6146	3,1446	1,7290	2,9798	1,7359	3,5690	2,5507
	Spinous process (L3); patient 2	3,5920	2,4678	3,6024	2,6166	3,5084	2,6886	4,2114	2,9194	3,7442	2,8609

	Spinous process; on average	3,1932	2,1898	2,9079	2,1156	3,3265	2,3075	3,5956	2,3277	3,6566	2,7058
	Lamina of the vertebral arch (L3)	3,0307	1,8449	3,1620	1,7391	2,4366	1,7410	2,7725	1,6977	4,9436	3,1651
	Insertion-related tendinopathy of the left shoulder	4,7200	2,8195	4,7309	2,9810	4,3164	2,9379	5,6417	3,2775	4,3482	2,9042
	Articulatio capitis costae (7., right)	2,7592	1,9641	2,7812	1,8276	2,3891	1,8371	2,9675	2,0745	2,9413	2,0602
	Liposarcoma	0,7924	0,5742	0,6755	0,5251	0,9200	0,5946	0,5783	0,4473	0,8858	0,6583
FAPI-74	Right temp	5,0520	3,1051	5,2686	3,0615	5,1760	3,3932	5,0113	2,9701	6,1544	4,1220

	oromandibular joint										
	Left maxilla	5,1741	3,7227	4,3905	2,9743	4,6729	3,2939	5,0744	3,6663	4,1541	3,2869
	Spinous process (L1)	1,4673	1,0309	2,2970	1,5563	1,9933	1,5015	1,9729	1,5966	3,5126	2,5469

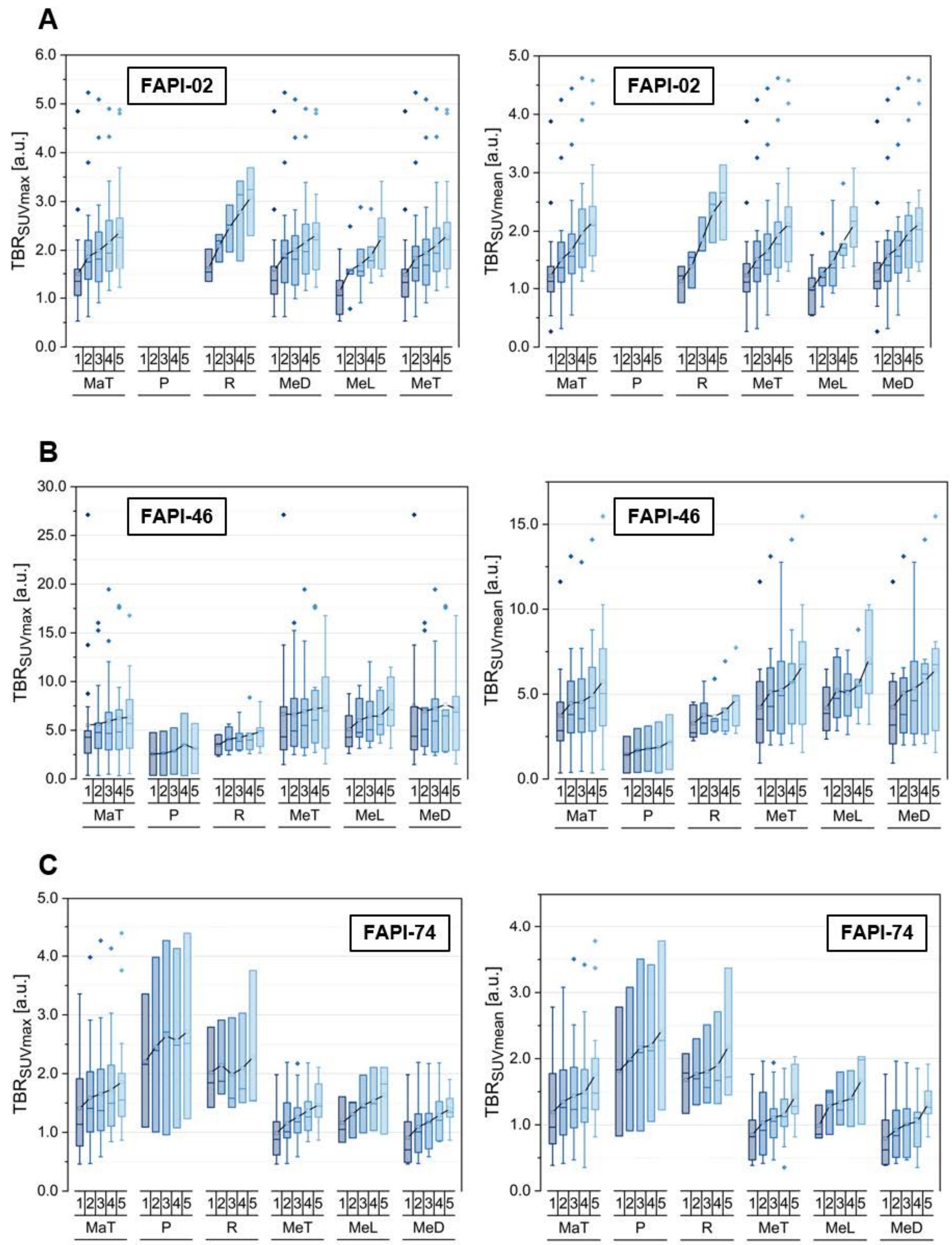

Supplemental Figure S1: Target-to-Background ratios (TBRs) for SUVmax and SUVmean values of pooled malignant (MaT) pathologies, including primaries (P), local recurrences (R), pooled metastases (MeT) with lymphogenic (MeL) and distant metastases (MeD), versus blood for the three ^{68}Ga-FAPI

tracer variants FAPI-02 (**A**), FAPI-46 (**B**) an FAPI-74 (**C**) over time with acquisition timepoints 10 min (1), 22 min (2), 34 min (3), 46 min (4) and 58 min (5) after injection.

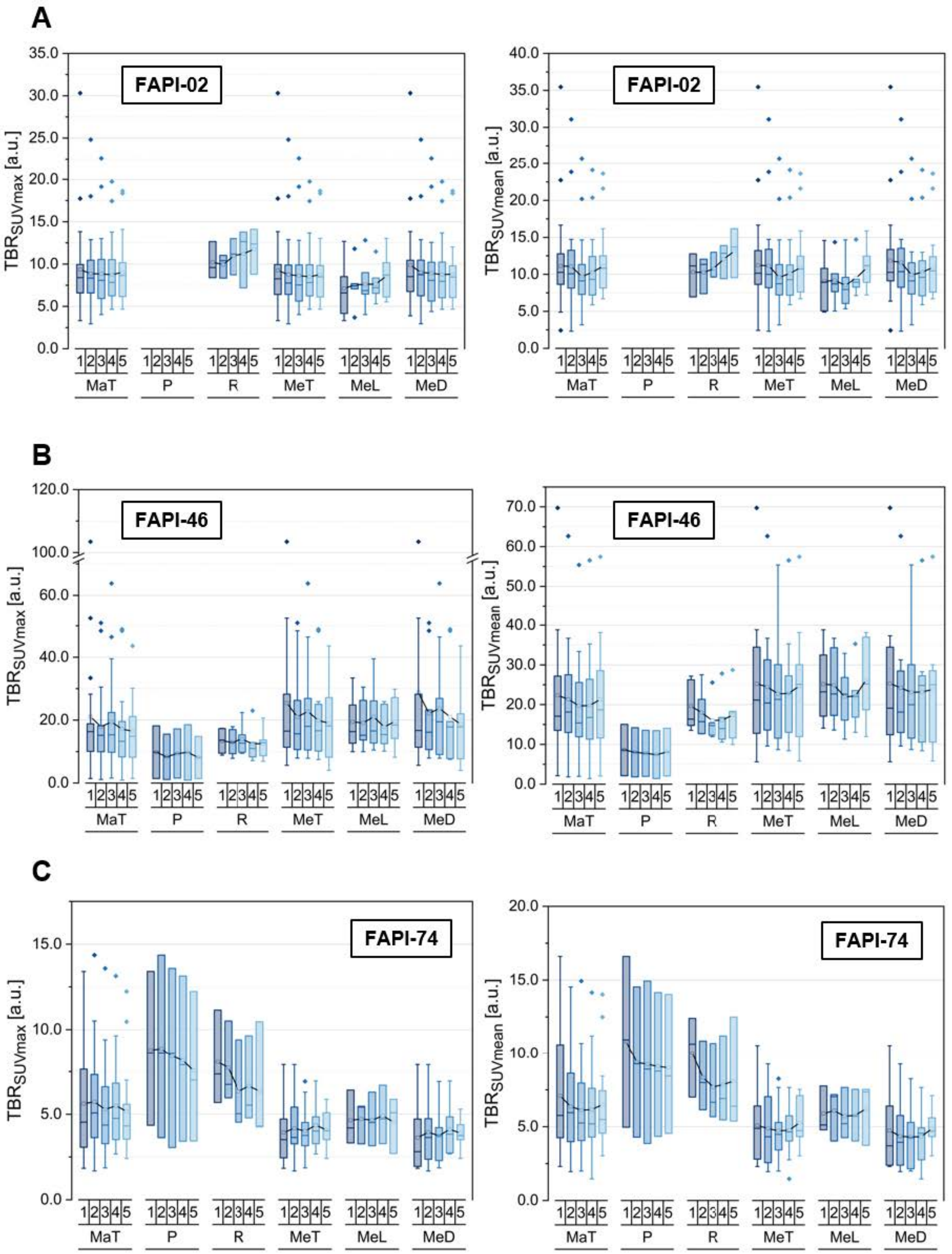

Supplemental Figure S2: Target-to-Background ratios (TBRs) for SUVmax and SUVmean values of pooled malignant (MaT) pathologies, including primaries (P), local recurrences (R), pooled metastases (MeT) with lymphogenic (MeL) and distant metastases (MeD), versus fat tissue for the three ^{68}Ga-FAPI

tracer variants FAPI-02 (**A**), FAPI-46 (**B**) an FAPI-74 (**C**) over time with acquisition timepoints 10 min (1), 22 min (2), 34 min (3), 46 min (4) and 58 min (5) after injection.

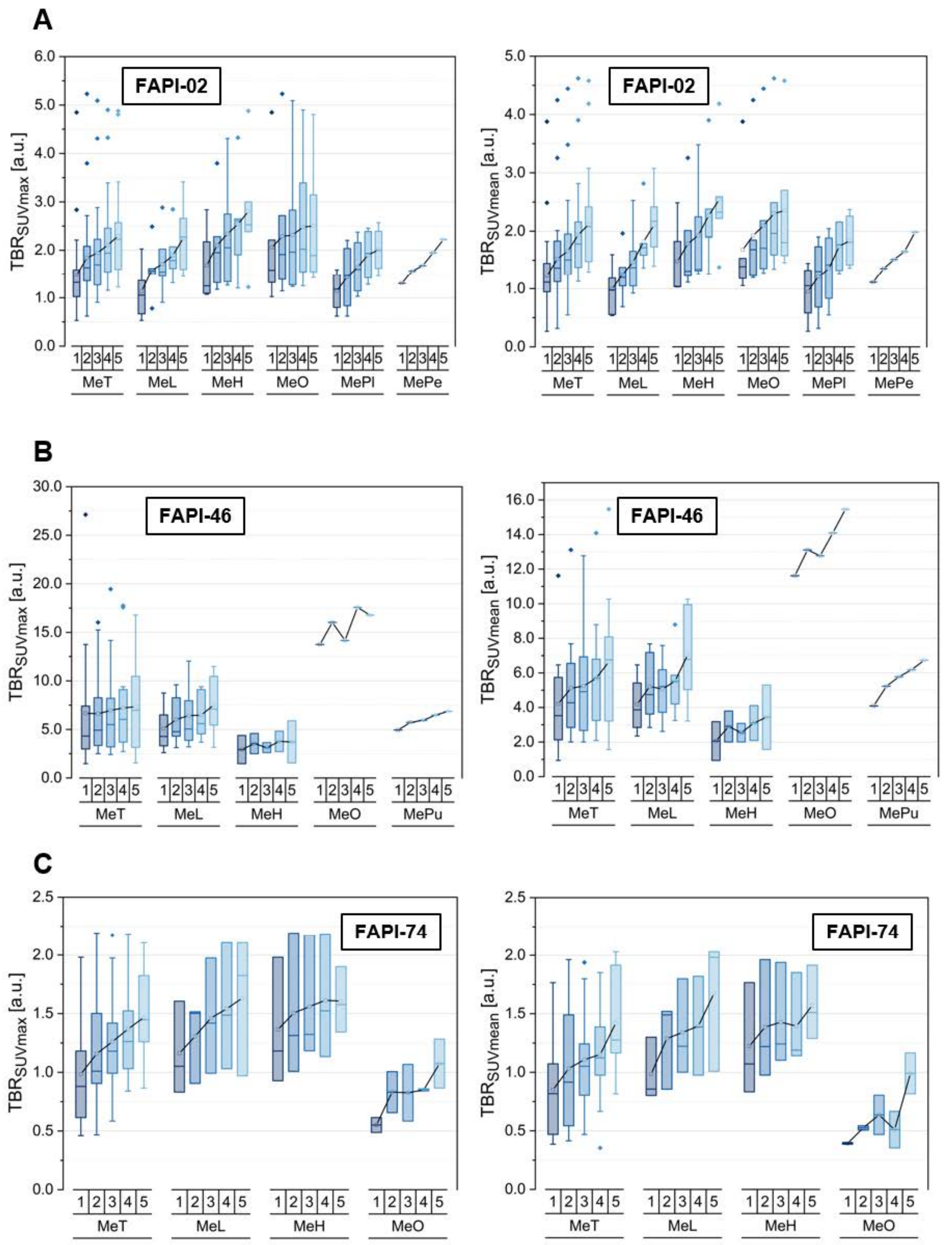

Supplemental Figure S3: Target-to-Background ratios (TBRs) for SUVmax and SUVmean values of pooled metastases (MeT) with lymphogenic (MeL) and distant metastases (MeD) including hepatic (MeH), osseous (MeO), pleural (MePl), peritoneal (MePe) and pulmonary metastases (MePu), versus

blood for the three ^{68}Ga-FAPI tracer variants FAPI-02 (**A**), FAPI-46 (**B**) an FAPI-74 (**C**) over time with acquisition timepoints 10 min (1), 22 min (2), 34 min (3), 46 min (4) and 58 min (5) after injection.

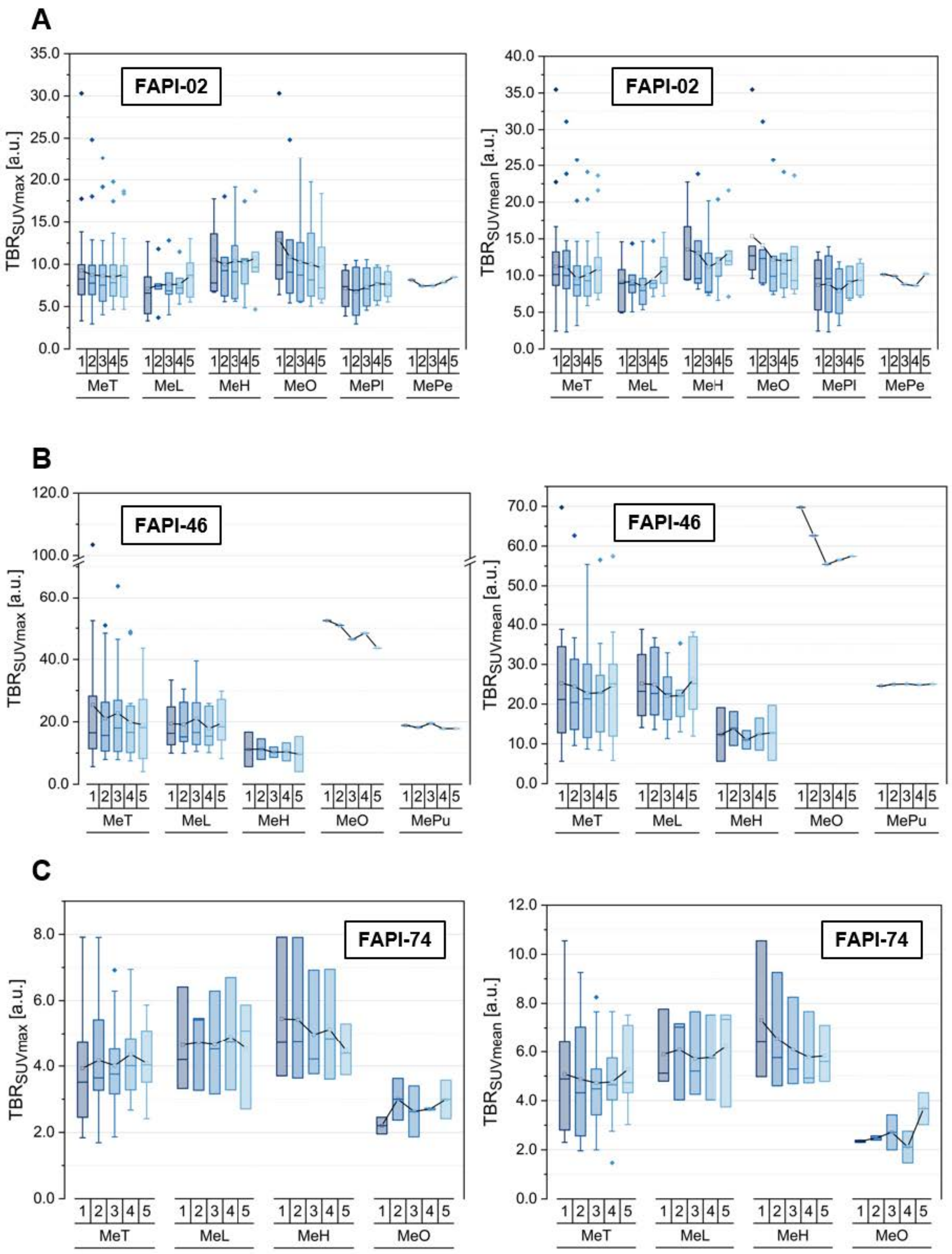

Supplemental Figure S4: Target-to-Background ratios (TBRs) for SUVmax and SUVmean values of pooled metastases (MeT) with lymphogenic (MeL) and distant metastases (MeD) including hepatic (MeH), osseous (MeO), pleural (MePl), peritoneal (MePe) and pulmonary metastases (MePu), versus fat

tissue for the three ^{68}Ga-FAPI tracer variants FAPI-02 (**A**), FAPI-46 (**B**) an FAPI-74 (**C**) over time with acquisition timepoints 10 min (1), 22 min (2), 34 min (3), 46 min (4) and 58 min (5) after injection.

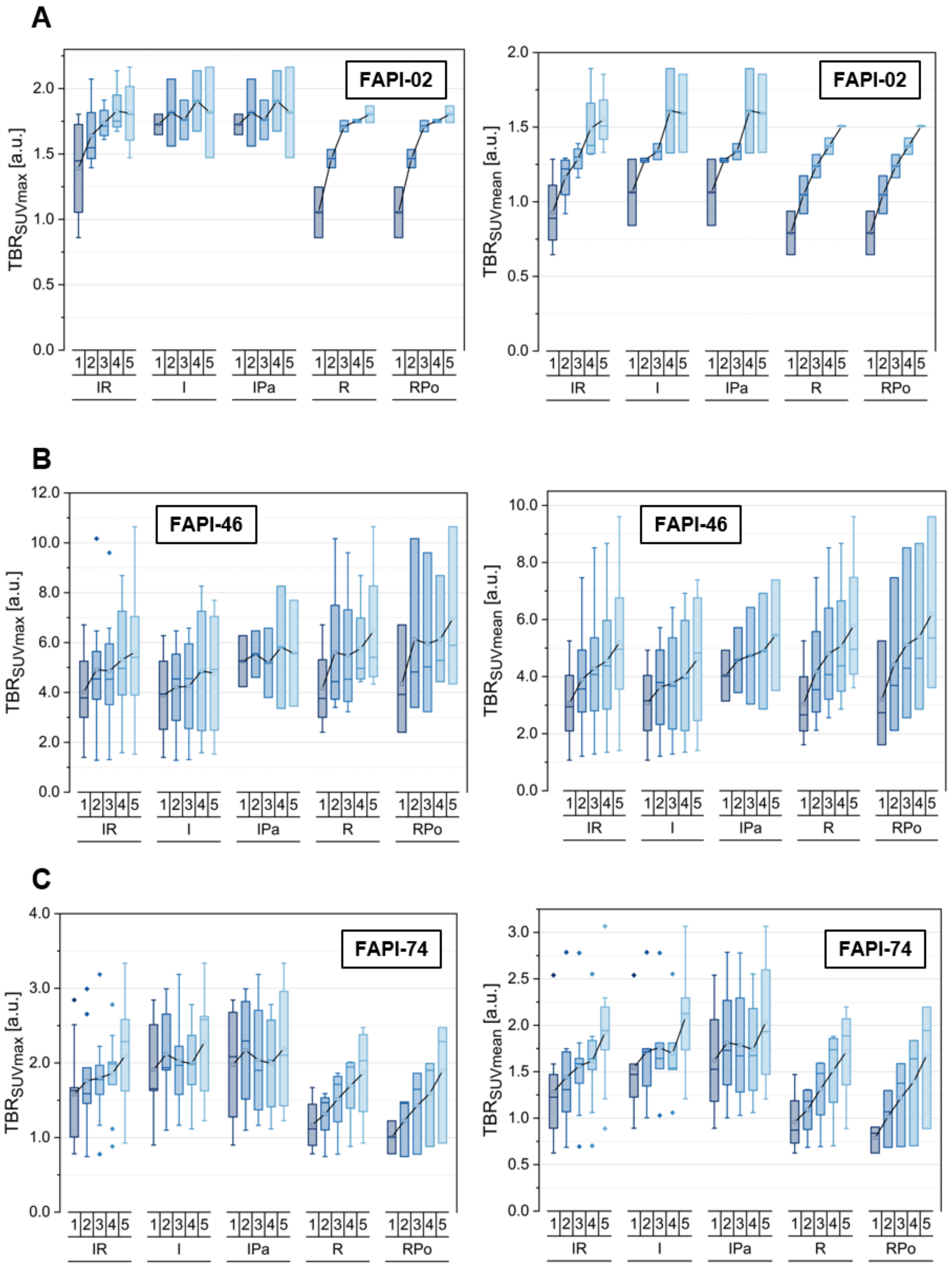

Supplemental Figure S5: Target-to-Background ratios (TBRs) for SUVmax and SUVmean values of pooled inflammatory/reactive lesions (IR), including inflammatory manifestations (I) with inflammatory lesions of the pancreas (IPa) and reactive manifestations (R) with postoperative lesions (RPo), versus blood for the three ^{68}Ga-FAPI tracer variants FAPI-02 (**A**), FAPI-46 (**B**) an FAPI-74 (**C**) over

time with acquisition timepoints 10 min (1), 22 min (2), 34 min (3), 46 min (4) and 58 min (5) after injection.

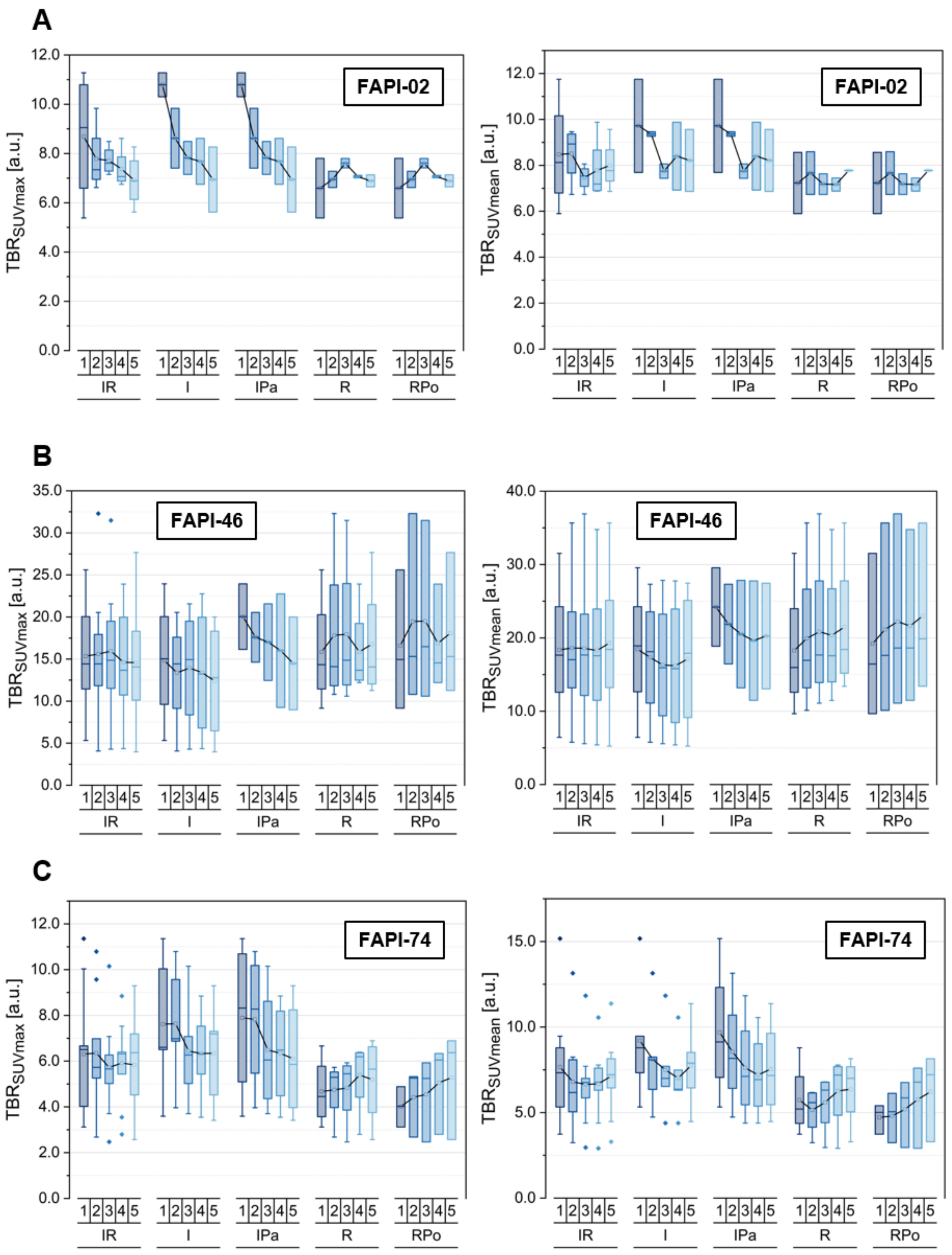

Supplemental Figure S6: Target-to-Background ratios (TBRs) for SUVmax and SUVmean values of pooled inflammatory/reactive lesions (IR), including inflammatory manifestations (I) with inflammatory lesions of the pancreas (IPa) and reactive manifestations (R) with postoperative lesions (RPo), versus fat tissue for the three ^{68}Ga-FAPI tracer variants FAPI-02 (**A**), FAPI-46 (**B**) an FAPI-74 (**C**)

over time with acquisition timepoints 10 min (1), 22 min (2), 34 min (3), 46 min (4) and 58 min (5) after injection.

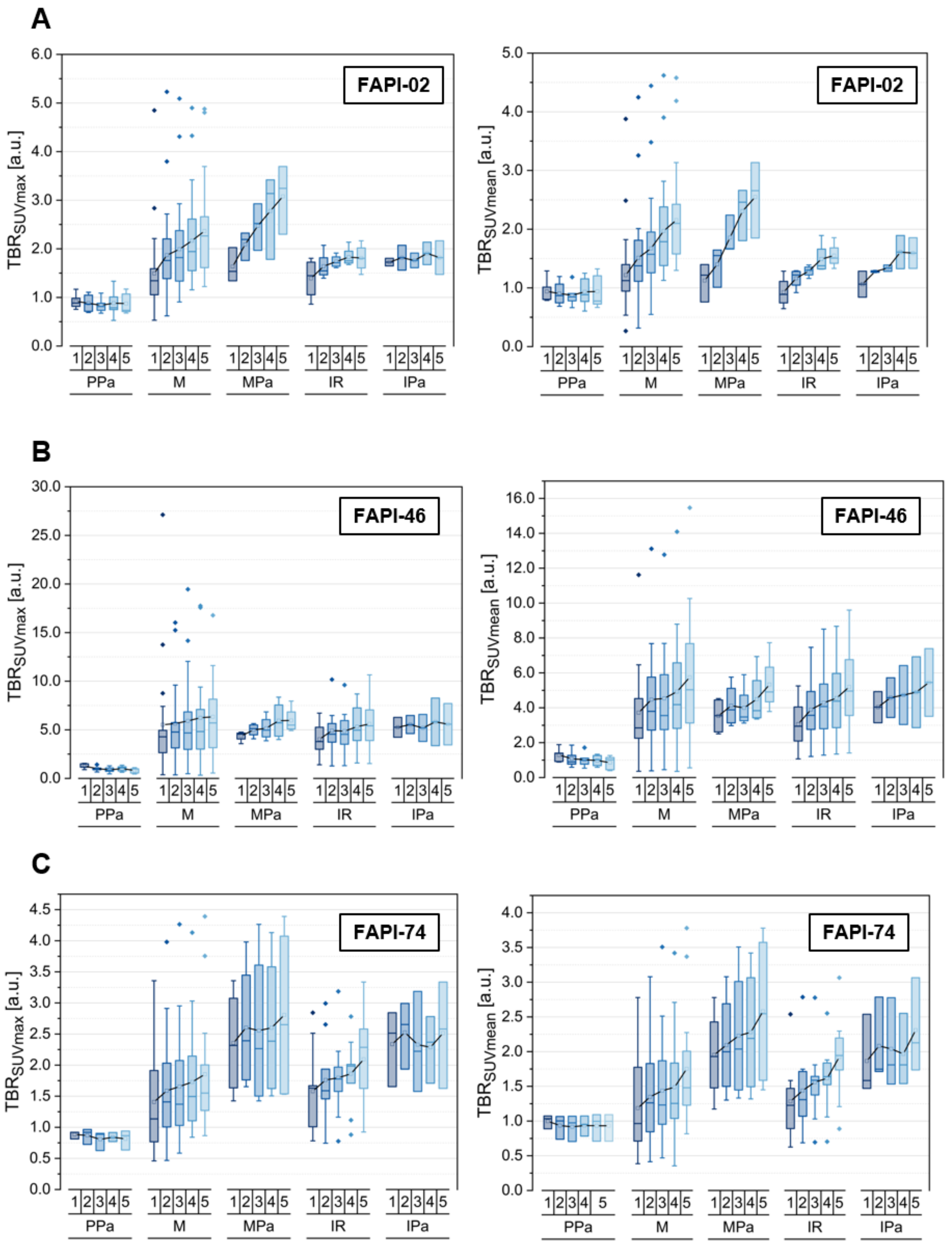

Supplemental Figure S7: Target-to-Background ratios (TBRs) for SUVmax and SUVmean values of the pancreas, including physiological pancreas tissue (PPa), pooled malignant lesions (M) with malignant lesions of pancreas (MPa) and pooled inflammatory/reactive lesions (IR) with inflammatory lesions of

pancreas (IPa), versus blood for the three ^{68}Ga-FAPI tracer variants FAPI-02 (**A**), FAPI-46 (**B**) an FAPI-74 (**C**) over time with acquisition timepoints 10 min (1), 22 min (2), 34 min (3), 46 min (4) and 58 min (5) after injection.

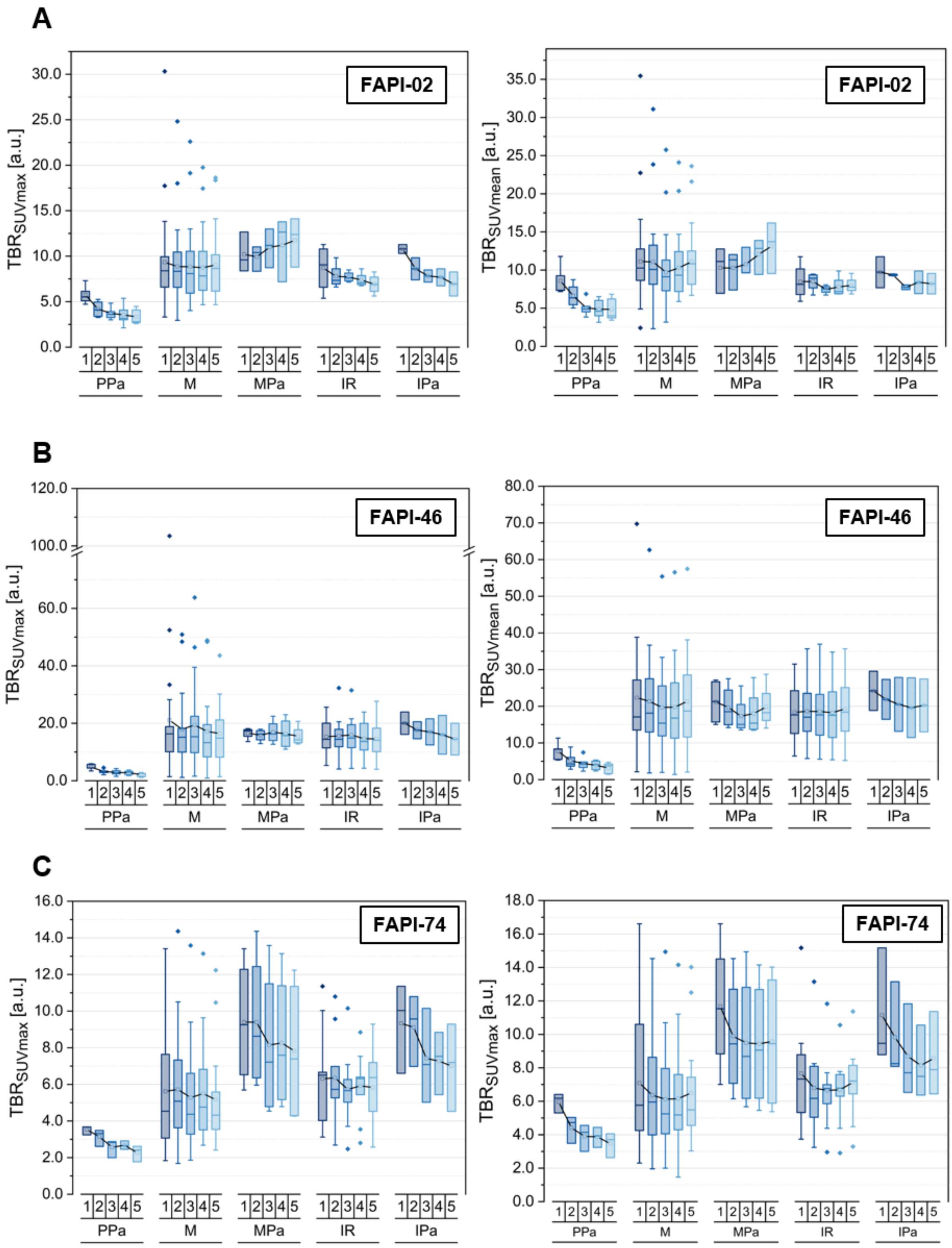

Supplemental Figure S8: Target-to-Background ratios (TBRs) for SUVmax and SUVmean values of the pancreas, including physiological pancreas tissue (PPa), pooled malignant lesions (M) with malignant lesions of pancreas (MPa) and pooled inflammatory/reactive lesions (IR) with inflammatory lesions of pancreas (IPa), versus fat tissue for the three ^{68}Ga-FAPI tracer variants FAPI-02 (**A**), FAPI-46 (**B**) an FAPI-

74 (**C**) over time with acquisition timepoints 10 min (1), 22 min (2), 34 min (3), 46 min (4) and 58 min (5) after injection.

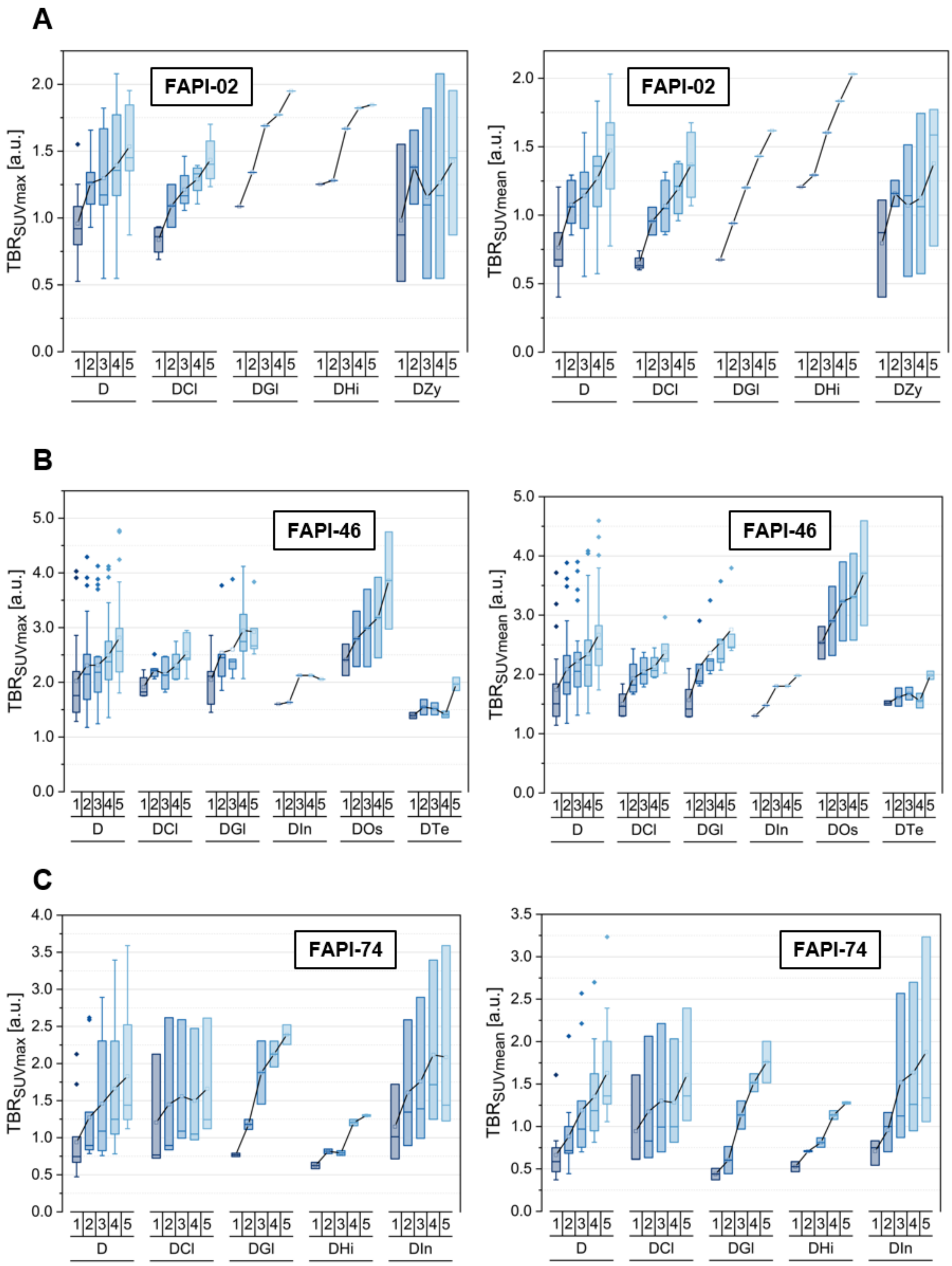

Supplemental Figure S9: Target-to-Background ratios (TBRs) for SUVmax and SUVmean values of degenerative lesions (D) located at acromioclavicular joints or sternoclavicular joints (DCl), at zygapophysial joints (DZy), glenohumeral joints (DGl), hip joints (DHi), osteophytes (DOs),

temporomandibular joints (DTe) and including insertion-related tendinopathy (DIn; located at collum femoris) versus blood for the three ^{68}Ga-FAPI tracer variants FAPI-02 (**A**), FAPI-46 (**B**) an FAPI-74 (**C**) over time with acquisition timepoints 10 min (1), 22 min (2), 34 min (3), 46 min (4) and 58 min (5) after injection.

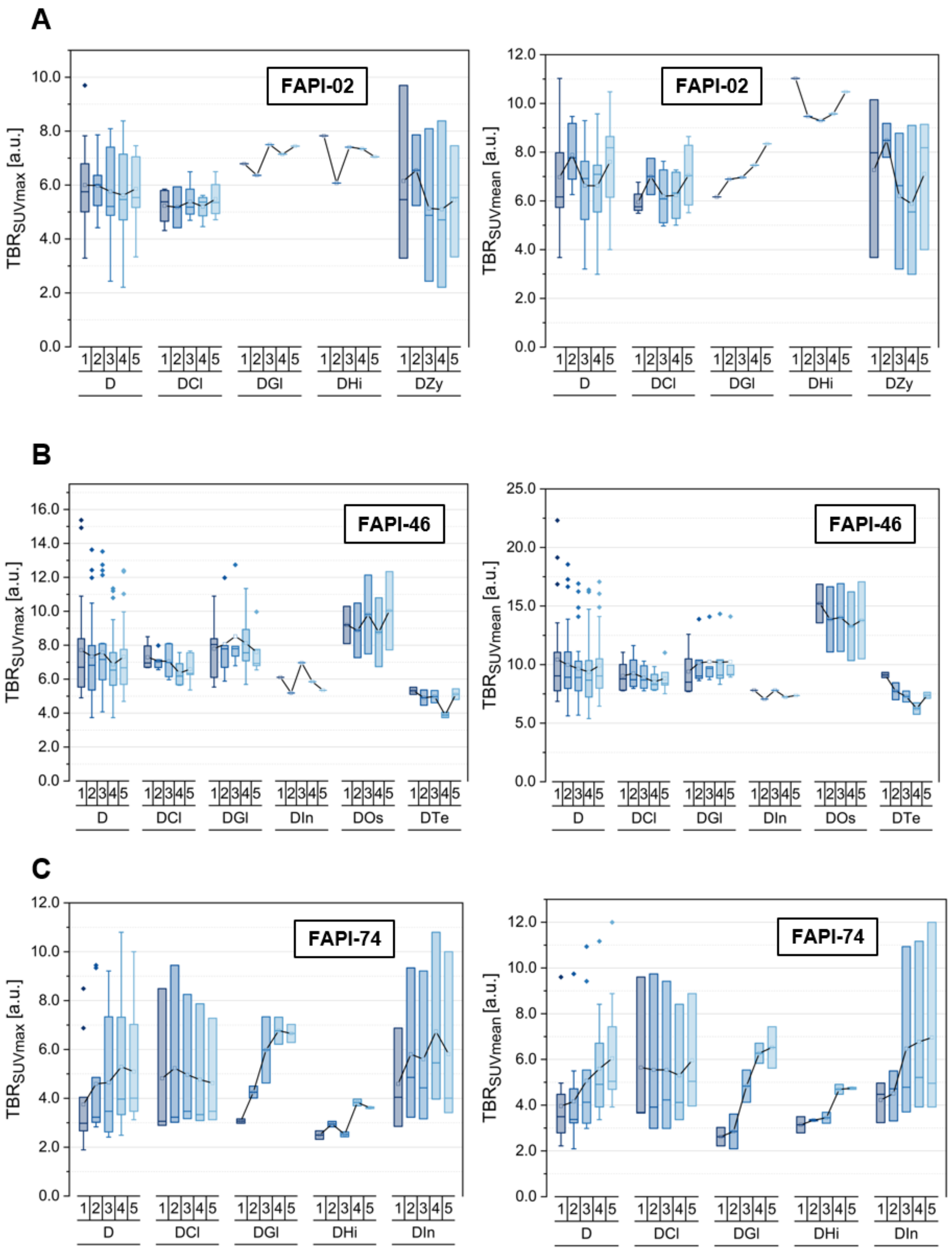

Supplemental Figure S10: Target-to-Background ratios (TBRs) for SUVmax and SUVmean values of degenerative lesions (D) located at acromioclavicular joints or sternoclavicular joints (DCl), at zygapophysial joints (DZy), glenohumeral joints (DGl), hip joints (DHi), osteophytes (DOs), temporomandibular joints (DTe) and including insertion-related tendinopathy (DIn; located at collum

femoris) versus fat tissue for the three ^{68}Ga-FAPI tracer variants FAPI-02 (**A**), FAPI-46 (**B**) an FAPI-74 (**C**) over time with acquisition timepoints 10 min (1), 22 min (2), 34 min (3), 46 min (4) and 58 min (5) after injection.

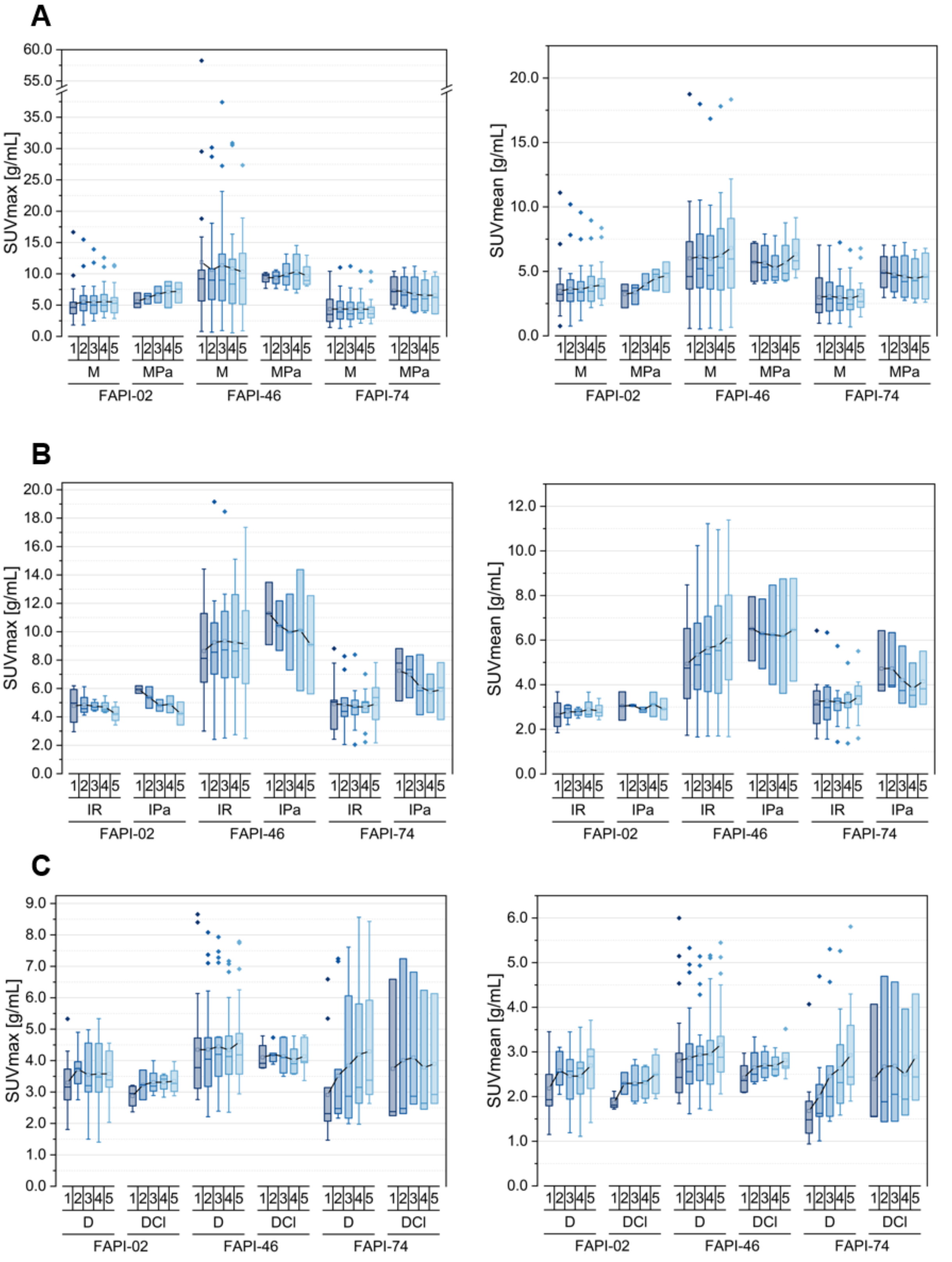

Supplemental Figure S11: Inter-tracer comparison of FAPI-02, FAPI-46 or FAPI-74 for the uptake in terms of SUVmax and SUVmean values regarding pooled malignant lesions (M) with malignant pancreatic lesions (MPa) (**A**), pooled inflammatory/reactive lesions (IR) with pancreatitis-associated

lesions (IPa) (**B**) and pooled degenerative lesions (D) with degenerative lesions located at acromioclavicular joints or sternoclavicular joints (DCl) (**C**) over time at 10 min (1), 22 min (2), 34 min (3), 46 min (4) and 58 min (5) after injection of ^{68}Ga-FAPI tracer. Boxes represent the interquartile range (IQR), whiskers the range of 1.5 IQR, horizontal line within the box indicates the median and small box the mean. Data outliers are shown separately within graph. Trending lines regarding mean are shown.

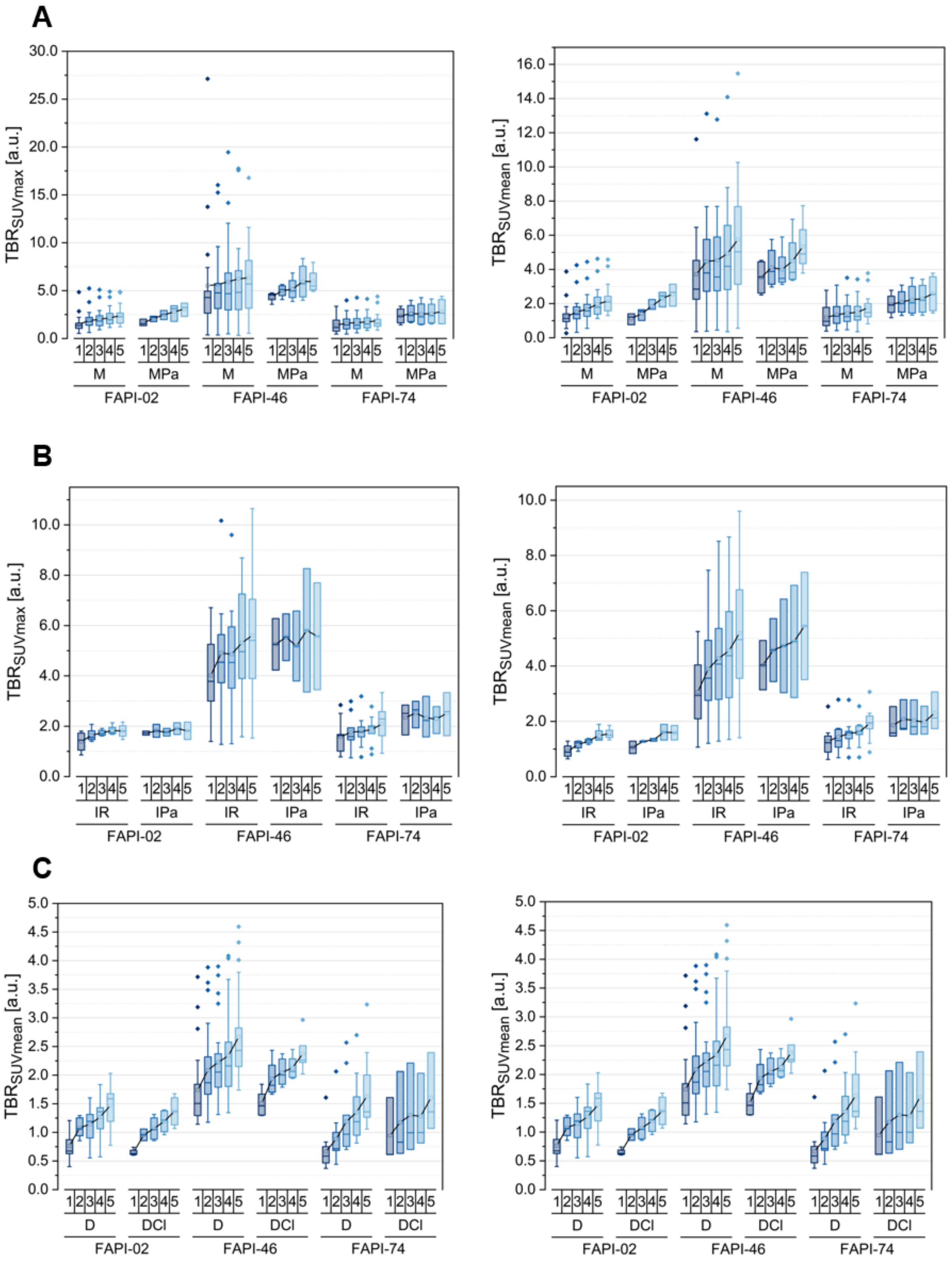

Supplemental Figure S12: Inter-tracer comparison of FAPI-02, FAPI-46 or FAPI-74 for the target-to-Background ratios (TBRs) for SUVmax and SUVmean values regarding pooled malignant lesions (M) with malignant pancreatic lesions (MPa) (**A**), pooled inflammatory/reactive lesions (IR) with

pancreatitis-associated lesions (IPa) (**B**) and pooled degenerative lesions (D) with degenerative lesions located at acromioclavicular joints or sternoclavicular joints (DCl) (**C**) versus blood over time with acquisition timepoints 10 min (1), 22 min (2), 34 min (3), 46 min (4) and 58 min (5) after injection of ^{68}Ga-FAPI tracer.

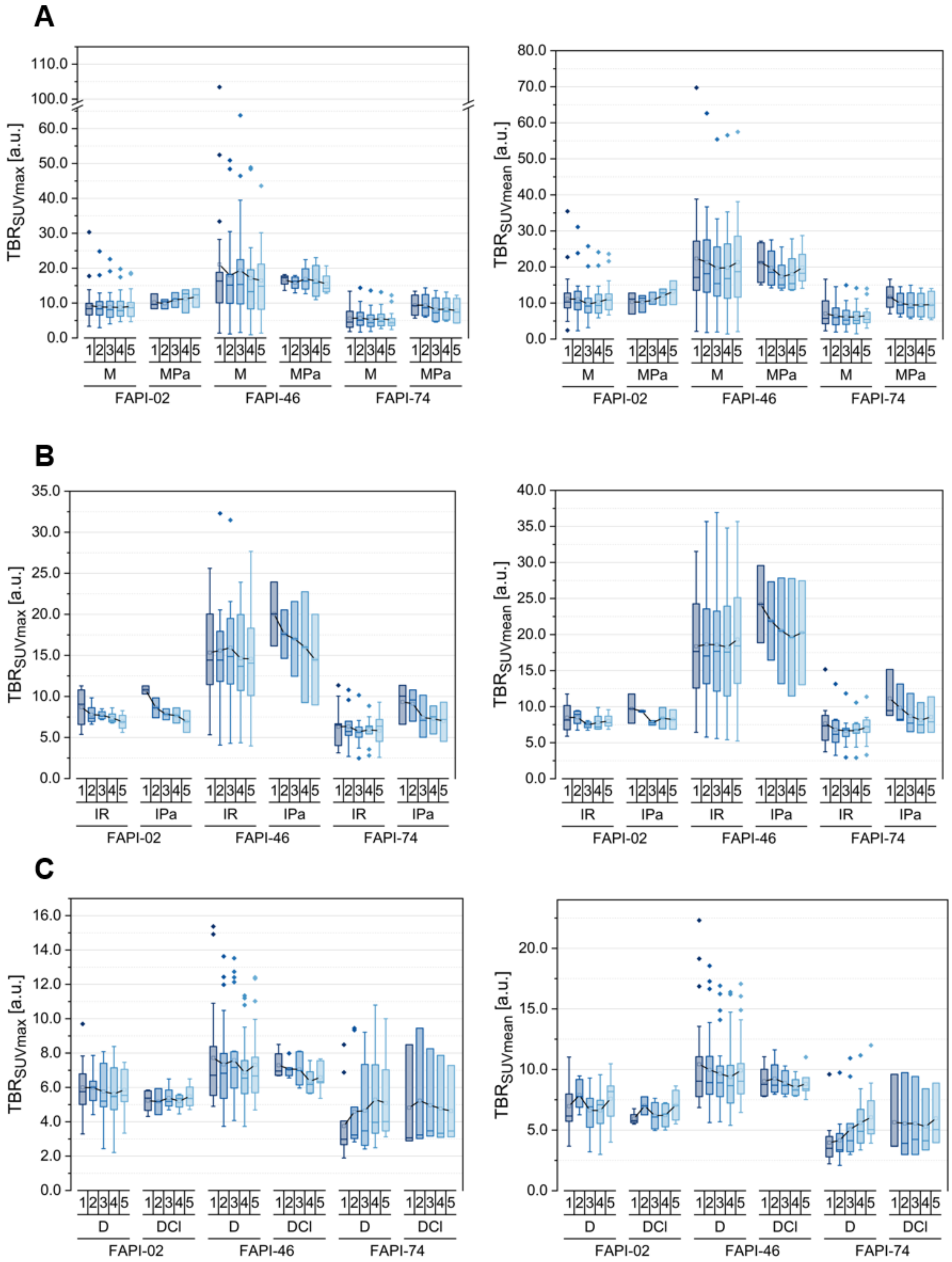

Supplemental Figure S13: Inter-tracer comparison of FAPI-02, FAPI-46 or FAPI-74 for the target-to-Background ratios (TBRs) for SUVmax and SUVmean values regarding pooled malignant lesions (M) with malignant pancreatic lesions (MPa) (**A**), pooled inflammatory/reactive lesions (IR) with

pancreatitis-associated lesions (IPa) (**B**) and pooled degenerative lesions (D) with degenerative lesions located at acromioclavicular joints or sternoclavicular joints (DCl) (**C**) versus fat tissue over time with acquisition timepoints 10 min (1), 22 min (2), 34 min (3), 46 min (4) and 58 min (5) after injection of ^{68}Ga-FAPI tracer.

Description of the results of Supplemental Figures S11,S12 and S13 regarding the inter-tracer comparison of uptake and TBRs over time

FAPI-46 showed the highest absolute values of uptake over time compared with FAPI-02 and FAPI-74. Respecting malignant lesions, approximately constant or only slightly increasing levels of uptake (SUVmax and SUVmean) were observed for all three tracer variants for the pooled malignant lesions as well as for the malignant lesions of the pancreas apart from the pancreatic malignant lesions using FAPI-02 manifesting a marked increase of uptake over time.

The TBRs versus blood, both TBR_{SUVmax} and $TBR_{SUVmean}$, increased over time with different gradients, whereas the TBRs versus fat tissue remained approximately constant or decreased slightly apart from the malignant lesions of the pancreas using FAPI-02, manifesting an increase over time.

Considering inflammatory and reactive lesions, both the pooled inflammatory/reactive and pancreatitis-related uptake using FAPI-02 decreased over time regarding SUVmax and remained unchanged respecting SUVmean. However, while the uptake of the pooled inflammatory/reactive lesions increased slightly over time (SUVmax and SUVmean) using FAPI-46, the pancreatitis-related uptake decreased (SUVmax) or remained constant (SUVmean) over time. Applying FAPI-74, the pooled inflammatory/reactive lesions showed an approximately constant level of uptake over time (SUVmax and SUVmean), whereas the pancreatitis-related uptake declined slightly over time. Again, the TBRs versus blood (TBR_{SUVmax}, $TBR_{SUVmean}$) increased over time at different slopes for the pooled inflammatory/reactive lesions and pancreatitis-related lesions using the three different tracer variants except the pancreatitis-related lesions regarding FAPI-02 showing constant uptake level over time for TBR_{SUVmax}. However, the TBRs versus fat tissue (TBR_{SUVmax}, $TBR_{SUVmean}$) manifested a marked decrease of TBRs for the pancreatitis-related lesions using the three different tracer variants, while the pooled inflammatory/reactive lesions showed a constant or only slightly decreasing TBRs over time.

Comparing the uptake behaviour of degenerative lesions using the three different tracer variants, slightly increasing uptake (SUVmax and SUVmean) was observed over time for the pooled degenerative and the specific degenerative lesions located at acromioclavicular joint or sternoclavicular joint for the three tracer variants except the pooled degenerative lesions using FAPI-74, resulting in a marked increase over time. The TBRs versus blood (TBR_{SUVmax}, $TBR_{SUVmean}$) increased over time for the pooled degenerative lesions as well as for the specific degenerative lesions located at acromioclavicular joint or sternoclavicular joint with different gradients, whereas the TBRs versus fat tissue featured a

heterogenous behaviour in a way that both TBR_{SUVmax} and $TBR_{SUVmean}$ showed approximately constant or only slightly decreasing curve progressions apart from the pooled degenerative lesions applying FAPI-74, which still increased over time.
